# WikiPathways 2024: next generation pathway database

**DOI:** 10.1093/nar/gkad960

**Published:** 2023-11-06

**Authors:** Ayushi Agrawal, Hasan Balcı, Kristina Hanspers, Susan L Coort, Marvin Martens, Denise N Slenter, Friederike Ehrhart, Daniela Digles, Andra Waagmeester, Isabel Wassink, Tooba Abbassi-Daloii, Elisson N Lopes, Aishwarya Iyer, Javier Millán Acosta, Lars G Willighagen, Kozo Nishida, Anders Riutta, Helena Basaric, Chris T Evelo, Egon L Willighagen, Martina Kutmon, Alexander R Pico

**Affiliations:** Data Science and Biotechnology, Gladstone Institutes, San Francisco, CA, 94158, USA; Maastricht Centre for Systems Biology (MaCSBio), Maastricht University, The Netherlands; Data Science and Biotechnology, Gladstone Institutes, San Francisco, CA, 94158, USA; Department of Bioinformatics - BiGCaT, NUTRIM, Maastricht University, The Netherlands; Department of Bioinformatics - BiGCaT, NUTRIM, Maastricht University, The Netherlands; Department of Bioinformatics - BiGCaT, NUTRIM, Maastricht University, The Netherlands; Department of Bioinformatics - BiGCaT, NUTRIM, Maastricht University, The Netherlands; Department of Pharmaceutical Sciences, University of Vienna, Austria; Micelio BV, Ekeren, Belgium; Maastricht Centre for Systems Biology (MaCSBio), Maastricht University, The Netherlands; Department of Bioinformatics - BiGCaT, NUTRIM, Maastricht University, The Netherlands; Department of Epigenetics. Van Andel Institute, Grand Rapids, MI 49503, USA; Department of Bioinformatics - BiGCaT, NUTRIM, Maastricht University, The Netherlands; Department of Bioinformatics - BiGCaT, NUTRIM, Maastricht University, The Netherlands; Unaffiliated, The Netherlands; Department of Biotechnology and Life Science, Tokyo University of Agriculture and Technology, Japan; Data Science and Biotechnology, Gladstone Institutes, San Francisco, CA, 94158, USA; Department of Bioinformatics - BiGCaT, NUTRIM, Maastricht University, The Netherlands; Department of Bioinformatics - BiGCaT, NUTRIM, Maastricht University, The Netherlands; Department of Bioinformatics - BiGCaT, NUTRIM, Maastricht University, The Netherlands; Maastricht Centre for Systems Biology (MaCSBio), Maastricht University, The Netherlands; Data Science and Biotechnology, Gladstone Institutes, San Francisco, CA, 94158, USA

## Abstract

WikiPathways (wikipathways.org) is an open-source biological pathway database. Collaboration and open science are pivotal to the success of WikiPathways. Here we highlight the continuing efforts supporting WikiPathways, content growth and collaboration among pathway researchers. As an evolving database, there is a growing need for WikiPathways to address and overcome technical challenges. In this direction, WikiPathways has undergone major restructuring, enabling a renewed approach for sharing and curating pathway knowledge, thus providing stability for the future of community pathway curation. The website has been redesigned to improve and enhance user experience. This next generation of WikiPathways continues to support existing features while improving maintainability of the database and facilitating community input by providing new functionality and leveraging automation.

## Graphical Abstract

**Graphical Abstract:**
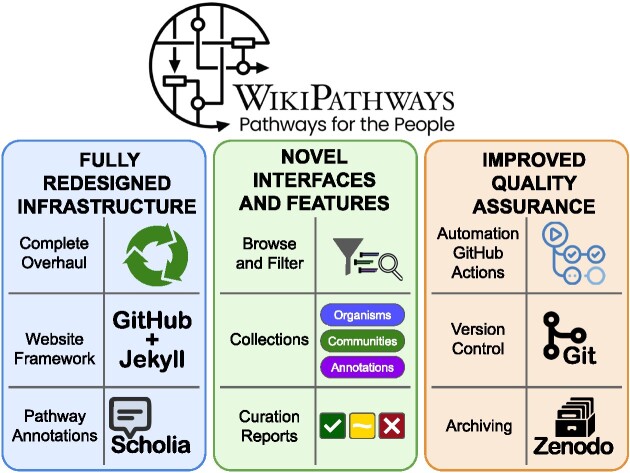

## Introduction

Since our last update in 2021 (1), there have been major developments to improve the long-term sustainability of WikiPathways, from infrastructure to user interfaces, leading to a renewed and restructured approach to sharing and curating pathway knowledge. Launched in 2007 as a research project to see if the technology behind collaborative text editing (*à la* Wikipedia) could effectively be applied to the curation of pathway models by the research community (2), WikiPathways has met with both success and challenges. To date, the efforts of 906 individuals via 46 923 edits have come together in WikiPathways to produce a unique, robust and now widely adopted pathway database. Taking the lessons we have learned along the way and in the face of growing technical debt associated with maintaining aging software and infrastructure, we implemented a plan to overhaul the WikiPathways system using free-tier, minimal-maintenance services in order to allow us to focus more on human-centered activities such as biocuration and community outreach.

In this article we provide an update on the pathway content and how it can best be utilized by communities of researchers, authors and programmers, all in the context of the new features and systems we are deploying in the evolving WikiPathways project.

## Content and general updates

**Figure 1.**
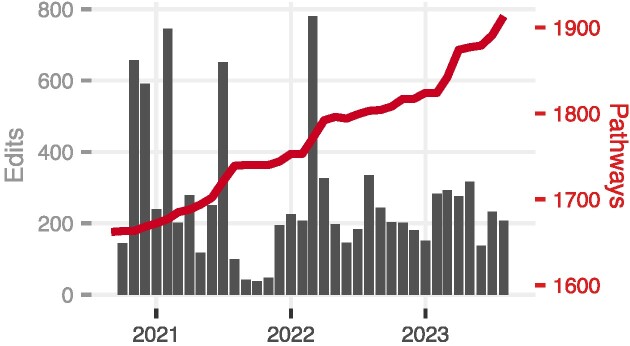
Recent growth of WikiPathways. The bar plot shows the total number of monthly edits, the line plot shows the total number of pathways in WikiPathways, and the x-axis is years. A dynamically updated version of this plot is available on the front page of the WikiPathways website. Note that the pathway count only includes human-curated content that has been reviewed and merged into the WikiPathways database; we are not counting inferred, converted or incomplete pathways.

Since our last update, the WikiPathways database has continuously grown, with an average of 84 new, original pathways per year (Figure 1). WikiPathways contains a total of 1913 human-curated and reviewed pathways for 27 species. As a result of our unique approach to community curation, the content at WikiPathways acquired 10 873 edits by 201 pathway authors in just over the past three years. With these achievements, WikiPathways now holds 36 334 gene products, 7052 metabolites and 85 647 interactions in total across all pathways. Nearly half of our pathways (866/1913) describe human biology. These pathways are automatically translated by homology mappings to nine other vertebrate model organisms. This process generates a secondary collection of 22,790 pathways which can be used in enrichment analysis, data visualization and further curation as species-specific models that can then be submitted to the main, manually curated collection at WikiPathways, github.com/wikipathways/wikipathways-homology. Monthly data releases dating back twelve months are available at data.wikipathways.org, providing snapshots of the pathway collection in a variety of data and image formats.

Beyond citations to previous WikiPathways journal articles, we have identified 1228 mentions of a total of 582 unique WikiPathways pathway model identifiers, e.g. WP4846 (3), in PubMedCentral articles over the past 13 years. Our content is shared in various reusable formats (e.g., GPML, GMT, SVG, RDF) to increase the usability and impact of pathway models. Additionally, we have established permanent archiving at Zenodo (zenodo.org/communities/wikipathways/search), where our collections are assigned citable DOIs, e.g. 10.5281/zenodo.8248445 for the August 2023 release of the human GMT files, to support researchers in providing accurate provenance for their data analysis pipelines and results. WikiPathways data is also included in popular tools such as the new gene set analysis feature in NDEx Integrated Query (4), the WikiPathways-specific functions enrichWP and gseaWP in the clusterProfiler R-package (5), and metabolomic and multi-omics data analysis with RaMP-DB 2.0 (6). Additionally, an increasing number of biological databases directly link to WikiPathways content, for example, links from the gene entries at GeneCards as of May 2022 (7). An overview of all tools and resources using WikiPathways content is available at tools.wikipathways.org.

### Pathways from the literature

We recently launched a complementary database called Pathway Figure OCR (available at pfocr.wikipathways.org). Facing the reality that the vast majority of pathway knowledge is still published as static images drawn using generic figure-making tools (despite our freely available pathway drawing tools), we developed a pipeline to specifically identify pathway figures indexed by PubMed Central via machine learning, and to extract genes, chemicals and disease terms by optical character recognition (OCR) (8). To date, we have identified over a hundred thousand pathway figures from the published literature and extracted a total of 4.4 million gene mentions across 638 organisms, over 300 000 chemicals and 40 000 disease term mentions. This collection includes 18 383 unique human genes from 71 423 pathway figures, which far exceeds the combined collections from WikiPathways, Reactome and KEGG. Pathway Figure OCR contents are available as GPML, TSV, JSON, R data files and markdown files and as GMT files for use in enrichment analysis. Targeting new and novel content, we have so far coordinated the curation of over two dozen pathway figures into fully annotated pathway models for WikiPathways (pfocr.wikipathways.org/browse/wikipathways.html), capturing all the relevant semantics intended by the original authors in a computational format. We encourage pathway authors to use these figures as starting points for new pathway models (see Updates for Authors and Reviewers for more details).

## New website

**Figure 2.**
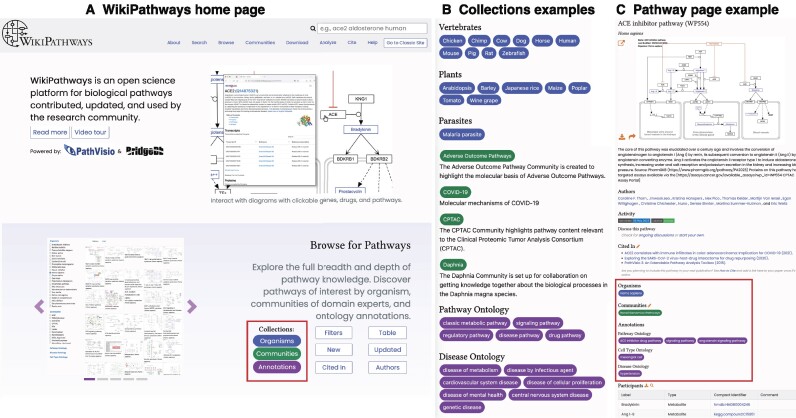
Composite screenshots of (**A**) the new WikiPathways.org home page and new cross-linking features via collections (**B**) throughout the new site, for example on pathway pages (**C**). Wherever organisms are mentioned, blue, rounded buttons are consistently used, establishing a visual lexicon and means of navigation. Likewise, green buttons represent our curator communities, and purple buttons signify ontology annotations.

Since our last update, WikiPathways.org has been completely overhauled and redesigned, from infrastructure to interface. The top of each page presents a search box, which is by far the most popular feature on the site, and an abbreviated sitemap of common pages (Figure 2).The home page begins with an introduction and link to a video tour (youtu.be/oSyoDU2r4Q0) of the new website produced by the TogoTV team of the Database Center for Life Science (doi.org/10.7875/togotv.2023.063). Following an introduction and example pathway page, four main sections offer galleries of images and links targeting how to browse, contribute, analyze and download WikiPathways content. The new search tool supports any keywords found in a pathway’s title, identifier, description, organisms, last edited date, ontology annotations (9) and sets of gene and metabolite labels. In addition, we currently offer myriad ways to browse pathway content, including by organism, community, ontology annotation, author, citation and new or updated status, as well as by interactive tabular and faceted search views. The sections for analysis and download offer a consolidated listing of information about data formats, programmatic access and compatible software and web tools. The section on how to contribute encourages all users to look into our self-paced WikiPathways Academy training system and highlights the current ‘Reviewer of the Week.’ The site-wide footer section contains a variety of project status badges and a more complete sitemap.

Leveraging the simple yet powerful concept of collections inherently supported by the Jekyll software, we can define sets of pathways by their annotated organisms and ontology terms from pathway, disease and cell type ontologies, as well as by the communities and authors involved in their curation. These collection types can then be used throughout the site to cross-link, browse and filter pathway content (Figure 2).

Ultimately, the most critical content is contained in the dedicated pathway pages that we host for each pathway model. The pathway page has been thoroughly redesigned, taking into consideration over a decade of user feedback and interactions with the prior versions of the site. For example, the title, identifier, organism, image and description fill the initial view. Without any scrolling, users have access to full screen and multiple download and sharing options. Further down, in order, users will find the site-wide, cross-linking elements for authors, organisms, communities and ontology annotations. New features include activity badges (e.g. last edited date and curation status), discussion options and links to any articles that cite the given pathway. The pathway page offers a table view of the molecular participants of a pathway, including label, type, compact identifier hyperlink and comments, as well as a hyperlinked bibliography, both of which can be downloaded as TSV files. See ‘New website features’ and Figure 4 below for more details.

**Figure 3.**
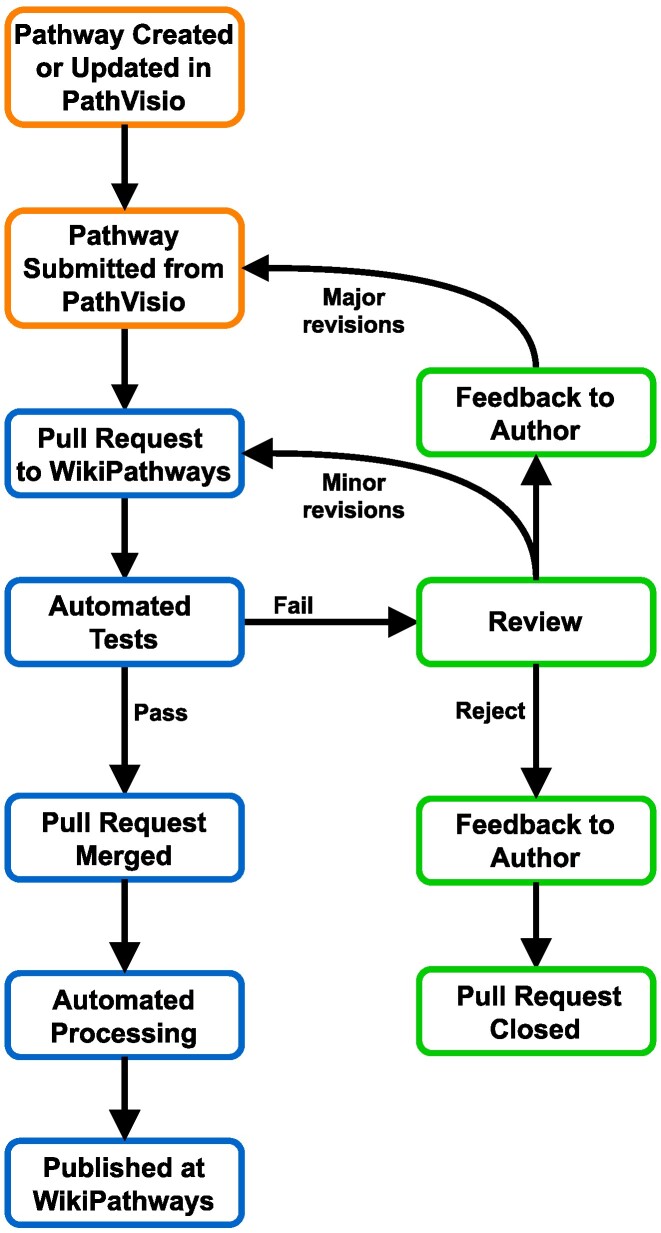
New pipeline for pathway submission, review, and acceptance. Orange boxes represent pathway author action; blue boxes represent automated steps; green boxes represent tasks performed by human reviewers.

**Figure 4.**
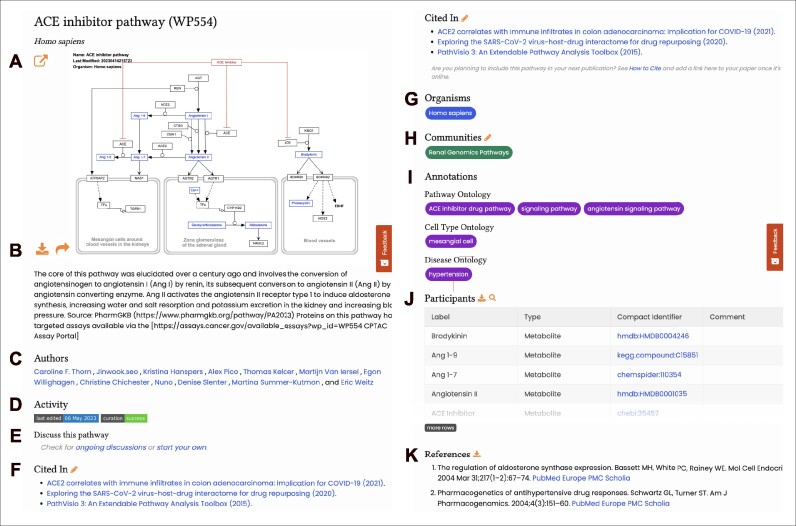
(A–K) An example of a pathway page on the updated WikiPathways website. Labels A–K highlight the features and interaction options on the page. This interface displays a button to open the model fullscreen (**A**), download and share the pathway (**B**), the authors (**C**), editing history and last activity (**D**), pathway discussion options (**E**), publications citing this pathway model (**F**), organisms relevant for this pathway (**G**), communities involved in this model (**H**), disease and pathway ontology annotations (**I**), participants of this pathway (**J**) and references to support this model (**K**).

In addition to modernizing the user interface of the website, our motivation for the change was the long-term sustainability of the project, and our approach as summarized in the following sections may hold lessons for other developers and maintainers of scientific databases.

### Novel infrastructure

The migration can be summarized as a set of swaps: in terms of infrastructure, we swapped out Mediawiki for Jekyll and GitHub. We have engineered a novel Jekyll-based wiki platform leveraging GitHub repositories together with GitHub Pages and GitHub Actions. This move allowed us to substitute the cost and burden of local and paid-tier cloud hosting for free-tier GitHub Pages and repositories. WikiPathways now runs with zero infrastructure costs and zero hardware concerns. In terms of maintenance and other ‘DevOps’ tasks, we swapped out self-managed (inconsistent) updates and (laborious) deployment for professional services (free-tier SaaS, PaaS and IaaS) and robust automated tooling (GitHub Actions) for highly customizable continuous integration and deployment (CI/CD). Finally, in terms of software development, we have swapped out PHP and SQL for HTML, JavaScript and flexible language choices, reflecting the change in culture and preferences within our developer team and applicant pool. Each GitHub Action is self-documenting using our named jobs and steps, which helps contributors add new functionalities to our open-source project, as well as inform developers on our current operating procedure. Pathways are still stored in the established Graphical Pathway Markup Language (GPML) format.

### Modern collaboration protocols

Coupled with the infrastructure migration to GitHub, WikiPathways now applies best practices from collaborative code management directly to the curation of pathway information. Version control via git, for example, together with pull requests (PRs) provides the core components of the curation process, enabling review, merging and rejection. Further, the pathway review performed by humans is significantly extended by incorporating automated tests, allowing for minor edits to be automatically merged, and freeing up time for human reviewers (previously called curators) to focus on issues requiring biological knowledge, judgment and care of the overall pathway. See the section on Updates for Pathway Authors and Reviewers for more details below.

### The pathway publishing pipeline

The pathway publishing process begins when a PathVisio (10) user chooses to submit their new or updated pathway model to WikiPathways (Figure 3). This action initiates a pull request (PR) to the wikipathways-database repository via GitHub’s API. Importantly, the pathway author does not need to know anything about git or PRs in order to perform these steps, and a free, general-purpose GitHub account replaces the need for WikiPathways-specific accounts. After submitting the pathway, several quality checks are performed to ensure that the pathway adheres to the WikiPathways guidelines and requirements for FAIR pathway models. First, a template checklist and series of tests are automatically applied to the pathway submission, leading to either a merge decision or the assignment of a human reviewer. This reviewer is selected from our Reviewer-of-the-Week roster, and they can decide to accept, reject, or address minor revisions on their own or advise the original author on major revisions required to accept the pathway model. Accepted changes automatically trigger a set of GitHub Actions that process the pathway model into each of the components needed to update the Jekyll site, perform identifier and homology mappings, prepare a bibliography and provide downloadable tables and image files. For a description of this pipeline from the perspective of pathway authors and reviewers, see the Updates for Authors and Reviewers section.

## Updates for biologists and chemists

The WikiPathways website is hosted using GitHub Pages. The new website offers a user-friendly interface and structure. New features have been added to the website to encourage interaction and collaboration from the scientific community. The front page clearly shows the different ways in which users can interact with our content (keyword search, download, or analyze pathways) and learn how to contribute through the WikiPathways Academy.

### New website features

Advanced search functionalities allow the user to search for pathways by keywords in the title and description, or by gene names, metabolites, organisms, ontology annotations, pathway identifiers (WPIDs) and year of last edit. Using the autocomplete function and search filters, the first 40 pathway results are displayed as the user types keywords in the search bar. Multi-term queries are supported by the new website, applying AND logic across multiple keywords.

The browse functionality enables the user to explore the full breadth and depth of pathway knowledge. This function can be used to discover pathways of interest by organisms, communities of domain experts and ontology annotations. The user can also explore the list of the latest pathways added, updated or cited. The ‘Filters’ button navigates to a gallery or list view of all WikiPathways pathways, with filter options at the disposal of the user to investigate the biology of interest. The ‘Table’ button provides similar filtering options, in one tabular overview.

Similar to the previous website, the new website has a dedicated page for each pathway (Figure 4). Based on feedback from WikiPathways users and professional designers, the pathway pages have been revamped to display the most relevant information in a discernible and interconnected manner. The top of each pathway page provides interactive viewing, linkouts, downloads and sharing options, including click-to-copy citations, permalinks and embed codes, as well as social media sharing (Figure 4A, B). Upon clicking on the pathway figure, a dedicated interactive view (the pathway viewer tool) will open in a new tab. This allows the user to zoom in and out, scroll through the figure and click on any annotated molecule node to open its Scholia page (11), which provides an innovative portal page generated from relevant Wikidata content. Each pathway further includes a list of pathway authors (Figure 4C), labels with the relevant organism (Figure 4G), communities (Figure 4H), pathway and disease annotations (Figure 4I), and references (Figure 4K). These pages also include a link to our novel representations of the edit history (powered by GitHub), discussions and issue trackers relevant to a given pathway (Figure 4D, E). All labels are rendered as buttons that are used consistently throughout the site. The user can click on a label button (e.g. *Homo sapiens*, Renal Genomics Pathways, ACE inhibitor drug pathway), and view all the pathways that have the same label. Furthermore, each pathway model is supported by a detailed curation report which incorporates the results of all automated quality control checks (mandatory and improvement points) shown as a success or fail status button (Figure 4D). The new pathway pages are designed to accommodate advanced options such as opening directly in NDEx (12) (Figure 4A) or querying drug interactions in Drugst.One (13) (Figure 4J, search icon). A list of all papers citing a given pathway (Figure 4F) is readily available on the pathway pages. Retaining wiki-like functionality, pencil icons throughout the site allow users to correct, edit and update details beyond just the pathway models themselves, for example about their citations of pathways, their author profiles and community details. Visitors can share their impressions of the new site along with feedback on specific parts of any page via red ‘Feedback’ buttons. The feedback widget provides users with the option to select an element of the page to include as a screenshot along with their written comments.

### Download options

The new pathway pages include the option to download the participants of a pathway (i.e. genes, proteins and metabolites) as an annotated TSV file with cross reference to various databases using compact identifiers (e.g. ncbigene:7040). These IDs are particularly useful to researchers wanting to copy, filter and merge lists of pathway genes, for example, in any number of provided identifier types (for genes and proteins, NCBI Gene, Ensembl, HGNC and UniProtKB; for metabolites Wikidata, ChEBI and InChIKey). The bibliography of references supporting a given pathway can also be downloaded as a TSV file. An overview of the available download options is provided at wikipathways.org/download.html.

### Analysis and visualization

Most commonly, WikiPathways pathways are used to perform functional enrichment analysis and generate publication-ready visualizations. Pathway information can be downloaded in various formats (e.g., GPML, GMT, RDF, SVG) to perform enrichment analysis using tools like Enrichr and WebGestalt. Software such as Cytoscape (14) and PathVisio (10) allow the direct import of WikiPathways pathway models to generate visualizations using user-friendly interfaces. We provide detailed and easy-to-follow tutorials on these tools wikipathways.org/analyze.html. A generous list of tools that make use of WikiPathways content or provide novel ways to query and display our collection of community-contributed pathways is available at tools.wikipathways.org.

### How to cite

To cite a specific pathway, the user can provide a direct link to the pathway page using a permanent URL in the format of wikipathways.org/instance/WP254. The permanent link of a pathway, as well as a full citation including pathway authors, is provided on each pathway page via the share pathway option to the left of the pathway figure. A major difference from the previous mediawiki-based page is that it is no longer possible to directly link to a specific version of the pathway using the revision number. Instead, the permanent link will always provide the latest version of the pathway. If it is necessary to link to a specific version, it is suggested to upload a PNG (and GPML) of the current version to Zenodo to retrieve a citable DOI. Once the citing paper is published, there is a new feature allowing users to add their publications in the ‘Cited In’ section of a pathway page.

## Updates for pathway authors and reviewers

### New website features for authors and reviewers

The new website has several improved author-related features. User registration has been streamlined, and only a general-purpose GitHub account is required. Author profile pages can be easily customized to include a biographical statement, affiliation, website and relevant links to external platforms such as GitHub, ORCID, Google Scholar and social media, e.g. wikipathways.org/authors/Egonw.html. The user’s GitHub avatar is automatically included on profile pages, along with the author’s communities of interest and the pathways they have contributed to as tabbed content, organized into ‘first-authored’, ‘co-authored’ and ‘list format’. Any mentions of authors on the new site (e.g., on individual pathway pages, listings of new or updated pathways) are automatically cross-linked to these customizable profile pages in order to highlight and acknowledge their curation contributions.

From the ‘Browse’ section on the main page, authors can quickly see new and updated content via prominent links to ‘New’ and ‘Updated’ pages where pathways are displayed as an image gallery or list of titles. From the same ‘Browse’ section on the front page, authors can navigate to the ‘Cited In’ page, which shows all pathways that have been cited in published literature. Adding a pathway to this list is easily done by editing the ‘Cited In’ section of the relevant pathway page.

On the pathway page, authors can access links and code to share and cite the pathway from the share icon directly under the pathway graphic. This interface provides direct links to share on various social media platforms and via email, as well as HTML code to embed the pathway image on a website. On the pathway page, the ‘last edited’ date is displayed in the ‘Activity’ section, along with any messages regarding curation issues. Automated curation includes tests for (among others) incorrect identifiers (e.g. for a different species), misformatted identifiers and using the metabolic conversion interaction arrow between protein DataNodes (with a list of exceptions). Identifier tests leverage the same BridgeDb identifier framework (15) that we use throughout WikiPathways and PathVisio. Curation status messages are linked to a customized report with aggregated results from a growing list of almost 100 specific curation tests (available at github.com/wikipathways/WikiPathwaysCurator). On the pathway page, the list of references cited in the pathway is now constructed with bibliographical metadata from external databases, allowing pathway authors to enter just an identifier in PathVisio (pending 4.0 release). This feature comes with support for references linked to a DOI, ISBN, or PubMed identifier, paving the way to support more types of publication identifiers e.g. PubMed Central, with linkouts to PubMed, Europe PMC and Scholia.

### Getting started

To help new authors get started and to organize curation efforts, we started a Pathway Curation Tasks repository (github.com/wikipathways/pathway-curation-tasks), where each task is described in a GitHub issue. Tasks are labeled with descriptive categories, to help authors identify tasks of interest. For example, the ‘good first issue’ label describes smaller tasks, such as suggested new pathways that are appropriate for beginners; the ‘needs work’ label is used for existing pathways that need improvement. Tasks can also be categorized by content and topic, for example, ‘drug mechanism pathway’, ‘plant pathway’ and disease-specific pathways. Any WikiPathways author can add a comment to a specific issue of interest to indicate that they are interested in working on a specific task.

**Figure 5.**
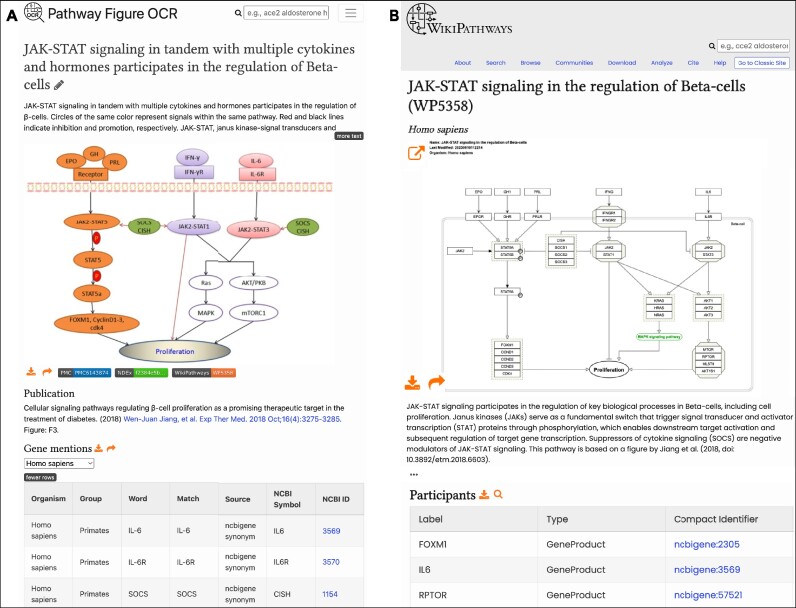
Side-by-side of original figure and model. (**A**) From the original figure (16), identifiable gene names were extracted by the Pathway Figure OCR pipeline, pfocr.wikipathways.org/figures/PMC6143874__etm-16-04-3275-g02.html. (**B**) The corresponding curated pathway model on WikiPathways, wikipathways.org/instance/WP5358 (17), which can then be used in a variety of downstream analyses and visualizations. The pathway model can be styled in Cytoscape (colors, shapes, labels, graphical annotations) to create a publication-ready pathway figure, for example to recreate the look of the original figure (see the section on Data Analysis and Visualization).

A new category of curation tasks is derived from the Pathway Figures OCR project (described in the ‘Pathways from the literature’ section), where figures from the literature have been identified as high-priority targets for pathway modeling (‘PFOCR’ label). Each figure has a dedicated page on the Pathway Figure OCR website (Figure 5A) from which the pathway genes and chemicals can be copied from their respective tables and pasted directly onto a pathway canvas in PathVisio as fully annotated nodes, thus serving as the starting point for a curated pathway model on WikiPathways (Figure 5B). In Figure 5, the example pathway modeling task involved specifying ambiguous labels such as ‘Receptor’ in the original figure, organizing the nodes in a meaningful layout and adopting standard graphical notations for complexes and interactions. Furthermore, the style of the resulting pathway model can be modified in Cytoscape to alter things like node color and shape, labels and graphical annotations, to create a pathway figure that is ready for publication. For more, see the Data Analysis and Visualization section.

### Pathway authoring and quality assurance

From the author’s perspective, the process of uploading and editing pathways via PathVisio will remain similar to the experience on the classic website. Authors submit a new or updated pathway from PathVisio, receive a status notification from the WikiPathways database repository (including feedback from a human reviewer if necessary), and then see their new content live at WikiPathways.org after their new pathway or edit is approved. The advantage of this approach is that it includes a transparent and orderly review process. The new website only hosts pathway content that has been reviewed and approved, rather than a mix of content at different stages of completion. This resolves the past confusion between content seen on the website and content used in analysis and official releases. The pathway editing tool, PathVisio, will guide users to either save their work in progress or upload their completed models that are ready for review. The review process has shifted from adding curation-status tags manually on the WikiPathways website to using proven standards for collaborative editing (e.g. pull requests, assigned reviewers). Collaborative, wiki-like pathway editing will continue to be the cornerstone of WikiPathways, now utilizing standards from source code management and the latest automation tools. New documentation, as well as self-paced learning via our WikiPathways Academy and expert curation workshops, are available for anyone interested in being a pathway author or reviewer at WikiPathways (described in Training Resources below).

From the reviewer’s perspective, our quality assurance process has been overhauled, leveraging git version control, pull requests (PRs) and automated merging to effectively reduce the burden on human reviewers. When an author makes edits to a pathway and requests a merger of their changes, all edits are summarized in a single PR with a template-based set of instructions and checklist items. The PR triggers a series of automatic checks to evaluate the content for basic quality parameters like the length, overlap and capitalization of a pathway’s title, and presence of a description, the completeness of data node identifiers, connected interactions and the presence of literature references. If the PR passes all checks, it is automatically merged. If the PR fails one or more checks, then a reviewer will be automatically assigned from the Reviewer-of-the-week roster, providing feedback for the author. PRs remain open for review, feedback and correction until all issues are resolved.

The Reviewer-of-the-week team is open to anyone and currently includes 16 reviewers from various research groups. In addition to monitoring content updates, reviewers also monitor a dedicated WikiPathways Help repository, github.com/wikipathways/wikipathways-help/discussions for questions from users. Each pathway page also includes a direct link to create a templated pathway-specific discussion in this repo to facilitate discussions. After initial review and response to questions, reviewers can choose to create a curation task from specific Help discussions in the Pathway Curation Tasks repository.

Monthly meetings are held for the WikiPathways reviewers to discuss a variety of curation-related topics. These meetings have proven instrumental in receiving feedback on our new website, our related tools (e.g., PathVisio and Cytoscape) and for refining user training materials, such as the WikiPathways Academy. We encourage any pathway author who is interested in joining the Reviewer Roster to contact us by email or discussion forum (github.com/wikipathways/wikipathways-help/discussions).

### Training resources

The WikiPathways Academy (academy.wikipathways.org) is the main training tool for WikiPathways and has an interactive design organized as a set of topic-centered tracks. The content is continuously updated, and we have added specific materials for modeling and drawing cellular compartments, transcription-translation, protein phosphorylation, molecular transport and using graphical lines to support adding legends. Along with the updated quality assurance procedures (described previously), a new track will be dedicated to training reviewers on this new system. WikiPathways.org includes a Help page (wikipathways.org/help) that summarizes the most important user-centered help topics. More in-depth topics are described in specific tutorials (e.g., cytoscape.org/cytoscape-tutorials/protocols/wikipathways-app) and an FAQ repository, github.com/wikipathways/wikipathways-faq/discussions.

### Communities

Fostering collaborations with diverse communities is a fundamental and integral component of the WikiPathways endeavor (1). Groups of users can come together to coordinate pathway-related activities focused on diseases, model organisms, consortiums and many more topics. The new website integrates communities better with their members and pathways of interest via the green Communities buttons used consistently across pages. Each community page displays the related pathways in a thumbnail grid and in a table view; buttons are available with direct links to Table and Filter views of the community content. Community members and authors of community pathways are listed on the main page for each Community, highlighting community members in a consistent and transparent manner.

Currently, there are 15 communities represented on WikiPathways. These include the COVID-19 pathway community portal (covid.wikipathways.org), which is a part of the COVID-19 Disease Map (18) initiative to create a repository of all molecular interaction diagrams and pathways related to COVID-19. Another example is the Adverse Outcome Pathways (AOP) Community (aop.wikipathways.org), which has utilized WikiPathways as a valuable integration tool to establish a seamless linkage between AOPs and the AOP-Wiki (aopwiki.org) containing detailed descriptions of measurements used in chemical risk assessment. This effort not only provides researchers with a more detailed understanding of molecular processes associated with toxicity, but also enables in-depth analysis of toxicological transcriptomic data, establishing direct connections between these insights and the Key Events integral to AOPs (19). These molecular AOPs are accessible via the AOP Community on WikiPathways, providing a complete view of toxicity mechanisms.

Pathway curation workshops offer an excellent opportunity to gather experts and enhance pathway models using WikiPathways. An illustrative case is a recently organized workshop centered around the rare cancer known as pleural mesothelioma, commonly linked to asbestos exposure. This collaborative endeavor culminated in the creation of a comprehensive molecular pathway (wikipathways.org/instance/WP5087), and subsequent journal publication, further advancing our understanding of the disease mechanisms (20).

## Updates for data miners and programmers

We continually work to provide WikiPathways content more accessible to data miners and programmers. Here we present the latest updates and highlights related to our new website, data accessibility and data analysis, which we think will facilitate various data mining and programming tasks.

### New website

Our new website now features direct links to essential resources, including data archives, SPARQL UI and documentation for both our web service API and client-side libraries in R and Python. These additions, alongside the advanced search, browse and download options mentioned earlier, provide efficient guidance for data miners and programmers to access WikiPathways content manually or programmatically. We also give direct access to JSON representations of the pathway contents (e.g. wikipathways.org/json/listPathways.json), enabling quick ingestion of all WikiPathways content into any pipeline. Moreover, each pathway page now has user-friendly options to embed, share and cite an individual pathway, aiming for easy dissemination and proper attribution.

### Data accessibility

We have developed various ways to access WikiPathways data over the years and our efforts continue to make it even more easily accessible. Here we summarize the existing methods of content retrieval, encompassing access via URL, SPARQL, web services, JSON, client-side libraries and Zenodo, and explain our plans to improve the accessibility further.

#### Access via URL

Our data archive (data.wikipathways.org) stores monthly releases of WikiPathways content (snapshots taken on the 10th of every month) in GPML, GMT, RDF and SVG formats. The releases in GPML, GMT and SVG formats can be downloaded via URL by using the following pattern:

https://data.wikipathways.org/date/format/wikipathways-date-format-species.extension

where {date} is in the format of YYYYMMDD such as *20230810*, {format} is one of *gpml*, *gmt* and *svg*, {species} is the species name of interest such as *Homo_sapiens*, and the {extension} is *zip* for GPML/SVG and *gmt* for GMT formats. For example, GPML files of *Homo sapiens* released in August 2023 can be accessed via the following URL: https://data.wikipathways.org/20230810/gpml/wikipathways-20230810-gpml-Homo_sapiens.zip.

On the other hand, RDF format allows users to download information about all species as a whole and uses the following pattern:

https://data.wikipathways.org/date/rdf/wikipathways-date-rdf-type.zip

where {type} is one of *wp*, *gpml* and *authors*. For example, all pathway information released in August 2023 can be accessed via the following URL: https://data.wikipathways.org/20230810/rdf/wikipathways-20230810-rdf-wp.zip.

For the latest releases, replace the file path {date} with *current* and access the .zip files per subfolder.

#### Access via SPARQL

WikiPathways content is available in semantic web format as Resource Description Framework (RDF), and can be accessed via an easy-to-use SNORQL interface (1), sparql.wikipathways.org. This interface includes eight categories of pre-written SPARQL queries that users can click on, execute, or even adapt for more customization. This allows various types of data queries to be done including but not limited to queries related to pathways, interactions, data sources and literature. For more advanced use cases, we provide a Virtuoso SPARQL query editor and endpoint at sparql.wikipathways.org. More information on using the query editor and making SPARQL queries inside some other programming languages such as Perl, Java and R can be found at rdf.wikipathways.org.

#### Access via web services and JSON

Our active support continues for web services and client-side libraries in R, Python and Java to provide easy programmatic access to WikiPathways content.

During our transition to the new site, we are maintaining our old web services in deprecated status while encouraging the adoption of our new services. The old web services use the following syntax to, for example, retrieve some general information about a pathway, such as name and species:

curl -X GET –header “Accept: application/json” “https://webservice.wikipathways.org/getPathwayInfo?pwId= WP554&format=json”

that returns the following response:

“pathwayInfo”: [

“id”: “WP554”,

“url”: “https://classic.wikipathways.org/index.php/Pathway:WP554”,

“name”: “ACE inhibitor pathway”,

“species”: “Homo sapiens”,

“revision”: “126205”]

However, since the database is not that large by modern standards, the new site can simply return a single JSON file with the information for all pathways, for example:

curl -X GET –header “Accept: application/json” “https://www.wikipathways.org/json/getPathwayInfo.json”

that returns the following (truncated) response:

“pathwayInfo”: [

“id”: “WP1”,

“url”: “https://www.wikipathways.org/instance/WP1”,

“name”: “Statin pathway”,

“species”: “Mus musculus”,

“revision”: “2022-12-27”,

“authors”: “Nsalomonis, MaintBot, Khanspers, BruceConklin, TestUser, AlexanderPico, Thomas, Mkutmon, Andra, Egonw, Ddigles, Eweitz”,

“description”: “Statins inhibit endogenous cholesterol production by competitive inhibition of HMG-CoA reductase (HMGCR), the enzyme that catalyzes conversion of HMG-CoA to mevalonate, an early rate-limiting step ...”,

“citedIn”: “” ,

...truncated... ]

The new approach supports bulk actions with better performance than the deprecated services, providing results for all pathways with a single query. The list of new JSON representations can be found at wikipathways.org/json. Until they are discontinued in 2024, the list of deprecated web service functions and their details can be found at webservice.wikipathways.org.

#### Access via client-side libraries

The client-side libraries in R (rWikiPathways), Python (pywikipathways) and Java (wikipathways-api-client-java) replace many lines of opaque code with single functions having easy-to-understand signatures for accessing WikiPathways content programmatically, e.g., getPathwayInfo(wpid). In addition to providing helper functions covering major web service and JSON use cases (see prior section), the scope of these libraries encompasses official monthly release files and easy-to-use GMT read/write functions. To illustrate, the helper function called getPathwayInfo can retrieve information for a specific pathway. For example, getPathwayInfo(‘WP554’) returns an R data frame containing id, url, name, species and revision.

rWikiPathways is routinely used by programmers, with more than 4000 downloads per year since 2021 (bioconductor.org/packages/stats/bioc/rWikiPathways). Similarly, pywikipathways is a Python package for programmatic access to WikiPathways content and provides the same function signatures as the rWikiPathways package. This allows for a seamless switch from R to Python without having to relearn how to use the package. We have also updated the Java library to use the latest versions of its dependencies, and we are currently working to enhance the library further by introducing new features. We will soon release this updated version under a new name: jWikiPathways. More information about rWikiPathways, pywikipathways and wikipathways-api-client-java can be found at bioconductor.org/packages/release/bioc/html/rWikiPathways.html, pywikipathways.readthedocs.io and github.com/wikipathways/wikipathways-api-client-java, respectively.

#### Access via Zenodo

Towards our efforts to enhance accessibility and citation of WikiPathways, we have extended our storage options. In addition to our existing data archive, we are now making monthly depositions in Zenodo (zenodo.org/communities/wikipathways). Zenodo provides a safe, trusted and easy-to-manage storage environment while also allowing each monthly released dataset to have its own version and a DOI. In this way, our users are now able to cite the specific monthly release of the WikiPathways content they utilized in their studies.

### Data analysis and visualization

Various tools and packages leverage WikiPathways content for data analysis and visualization purposes. Here, we highlight the utilization of WikiPathways content within a set of selected software packages that researchers use frequently. A complete list of tools that use WikiPathways alongside information on how they use it can be found at tools.wikipathways.org.

The *clusterProfiler* R package (5), which is a popular enrichment tool to analyze and visualize omics data, now has built-in support for enrichment analysis for WikiPathways pathway content via enrichWP and gseWP functions for Over-Representation Analysis (ORA) and Gene Set Enrichment Analysis (GSEA), respectively. Details of these functions can be found at yulab-smu.top/biomedical-knowledge-mining-book/wikipathways-analysis.html.

Enrichr (21) offers an easy-to-use interface for enrichment analysis with a large number of gene set libraries including WikiPathways. Several versions of WikiPathways content are available, with the latest version being named ‘WikiPathway_2023_Human’. Users can perform Enrichr enrichment analysis with WikiPathways content via either its web interface at maayanlab.cloud/Enrichr, R packages such as EnrichR (enrichr function) (cran.r-project.org/web/packages/enrichR) and rbioapi (22) (rba_enrichr function) or the Python package GSEApy (23) (enrichr function) to utilize Enrichr for enrichment analysis with WikiPathways content.

Additionally, the output for the enrichment analysis performed using the WikiPathways content can be visualized using customized Cytoscape visualization styles. The WikiPathways app for Cytoscape (24) provides easy access to pathway content from within the Cytoscape application (14). After installing the app, users can easily import GPML network files from WikiPathways. It also allows querying and importing pathways via the *Import* > *Network* > *Public Databases..*. interface provided by Cytoscape, or via the network search bar located at the top of the Network panel. These pathways can be imported either in their original diagram format or as a simplified network to support both data visualization needs and utilization in network analysis. More information about the WikiPathways App for Cytoscape can be found at apps.cytoscape.org/apps/wikipathways. Training materials are available as a tutorial (cytoscape.org/cytoscape-tutorials/protocols/wikipathways-app) and as a set of automation scripts for Python and R.

## Future plans

In the next few months, we plan to complete the transition to the new WikiPathways system. With the pending release of PathVisio 4, pathway models will be directly submitted as pull requests to the *wikipathways-database* GitHub repository, triggering the new review process (Figure 3). We will also be releasing a new GPML schema to capture additional pathway metadata with an improved pathway model. Development of the new site will continue as well, including adding an Interactions table to each pathway page that lists each annotated interaction, analogous to our current Participants table. Additional live edit capabilities will be enabled, such as making a quick edit to a pathway title, description, annotations, etc.

In the coming years, with the transition complete and our development and maintenance burden minimized, we will be focusing even more on human curation and curation support. Our new curation and review protocols will undergo iterative refinement as we gain experience with them. This will include the incremental addition of automated tests to substitute tedious and monotonous tasks for human reviewers, as well as new initiatives to recruit, welcome and empower pathway authors and reviewers. As a complementary effort, we also plan to further develop the Pathway Figures OCR database, capturing newly published pathway figures at a rate of over 1000 per month and extracting relevant entities (e.g., genes, proteins, chemicals, diseases) and relationships. This collection outpaces any other pathway database effort by a wide margin and is ideal not only for enrichment analyses (as gene sets) and machine learning (as a voluminous resource), but also as starting material for new, human-curated pathway content for WikiPathways.

Regarding all of the above, we plan to continue our outreach to tool developers and ensure high-quality pathway content is disseminated effectively in formats most useful to researchers.

## Conclusion

The WikiPathways database continues to grow with contributions from the community, and has become widely adopted for pathway information and visualization. Infrastructure changes using existing low-maintenance solutions like GitHub will enable automated pathway curation, increased focus on community outreach and continuous integration and delivery. The new WikiPathways website provides myriad features for interaction with the database, ranging from searching and browsing, to connecting with authors and communities, to making edits and learning how to contribute, to exporting pathway information in a variety of formats. We invite all interested researchers, authors and developers to participate in the WikiPathways project.

## Data Availability

WikiPathways is freely available at https://www.wikipathways.org/.
